# Nearly one-in-five mothers avoid colostrum in North Wollo Zone, Ethiopia: an institution-based cross-sectional study

**DOI:** 10.1017/jns.2021.97

**Published:** 2021-11-26

**Authors:** Misgan Legesse Liben, Nigus Bililign Yimer, Fentaw Wassie Feleke

**Affiliations:** College of Health Science, Woldia University, Woldia, Ethiopia

**Keywords:** Children, Colostrum avoidance, Ethiopia, Mothers, North Wollo, EDHS, Ethiopian Demographic and Health Survey, SPSS, Statistical Product and Service Solutions, TV, Television, WHO, World Health Organization

## Abstract

Colostrum contains antibodies that protect the newborn against disease. Despite this fact, many Ethiopian mothers see colostrum feeding as a cause of neonatal morbidity and mortality. These mothers believe that colostrum must discard to alleviate this effect. However, the cause of this misconception about colostrum was not well researched, particularly in this study area. The main aim of the present study was to assess colostrum avoidance and associated factors among mothers having children aged 6–59 months in North Wollo Zone, Northeastern Ethiopia. An institution-based cross-sectional study design was used. Descriptive statistics, binary and multivariable logistic regression analyses were used for the statistical analysis. The prevalence of colostrum avoidance was 19 % (95 % CI 15⋅03, 22⋅89 %) among mother–child pair aged 6–59 months. In multivariable logistic regression analysis, the most important predictors were breast-feeding initiation Adjusted Odds Ratio (AOR) 6⋅369; 95 %, Confidence Interval (CI) (3⋅067, 13⋅224), pre-lacteal feeding AOR 3⋅464; 95 % CI (1⋅721, 6⋅973), shared household decision about child feeding AOR 3⋅585; 95 % CI (1⋅563, 7⋅226), Index child sex AOR 2⋅103; 95 % CI (1⋅015, 4⋅358) and health facility delivery AOR 3⋅033; 95 % CI (1⋅293, 7⋅117). The colostrum avoidance in the present study was 19 %. The study recommends the promotion of institutional delivery, timely initiation of breast-feeding, the shared household decisions about child feeding, avoiding sex preferences and stopping pre-lacteal feeding were critically important.

## Introduction

Colostrum is a concentrated form of ‘immature milk’, which is very high in protein, antibodies, vitamin A and other protective components that are important for newborn^(1)^. According to the World Health Organization (WHO), colostrum is defined as the first milk, thick, sticky and clear to yellowish colour secreted during the first hour after birth up to 3 d. It is also called ‘liquid gold’ and ‘passport of life’ due to its highest content of antibodies as compared with mature breast milk^(2)^. It contains all the necessary nutrients for infants’ growth and development and antibodies that can protect from many childhood illnesses^(3,4)^. This nutrient is the most suitable food for the newborn, universally acknowledged as the perfect first food for infants and a suggested regimen for expressing and storing it during pregnancy including counselling on skin-to-skin contact in the first 24 h to maximise breast milk production for long term^(4)^. Neonates have a premature digestive system that outfits the small-volume rigorous form of the nutrient supply system of colostrum. The laxative influence of colostrum encourages the passage of newborn's first stool (i.e. meconium) which is important to prevent jaundice by removing extra bilirubin^(5)^.

The WHO approved the infant feeding strategy which included the recommendations for exclusive breast-feeding until 6 months of age with sustained breast-feeding during optimal complementary feeding practice. This recommendation has been endorsed by many countries including Ethiopia. Early and exclusive breast-feeding, including colostrum feeding is one of the most important things to achieve sustainable development goals by 2030 through improving infant survival^(6)^.

Globally, improving breast-feeding rates around the world could save the lives of more than 820 000 children under age 5 every year, the majority (87 %) under 6 months of age^(3,7)^. It is one of the optimal breast-feeding practice not only saves the lives of children under 5 years but also improves children's quality of life^(8)^. In resource-limited countries (i.e. Ethiopia), lactating women have limited understanding of colostrum advantages^(9–11)^. As a result, they preclude their infants from colostrum feeding immediately following delivery. Colostrum discarding removes vital nutrients which lead to high infant morbidity and mortality^(10,12–14)^. The prevalence of colostrum discarding varies from country to country and accounts for around 29⋅5^(10)^, 20^(15)^ and 11⋅2 % in India, Nepal and Cameroon, respectively^(16)^.

Different studies performed in different rural part of Ethiopia found that the prevalence of colostrum avoidance were 20⋅9^(17)^ and 6⋅3 %^(14)^ in Debre Markos and Aksum, respectively. Some societies considered colostrum as heavy, profuse, unclean, contaminated and risky for the health of infants. As a result, colostrum is thrown out, and honey, sugar water, glucose and water were given to the newborn as substitutes^(14)^. Even though there are some studies on the prevalence of colostrum avoidance practice in some parts of Ethiopia including the neighbourhood district called Kombolcha in South Wollo Zone and three districts of this study area with a result of positive predictors such as illness of index child, residence, counselling on timely initiation of breast-feeding, participation in pregnant women forum, husband employment and cultural beliefs with the prevalence of colostrum avoidance practice 11⋅4^(18)^ and 12 %^(19)^ with limited child age group with different study design. Still there has been a paucity of information about the level and determinant factors for colostrum avoidance which were not well studied mainly in this study area with the advantages of targeted age group. Therefore, the present study aimed to assess the level and predictors factors of colostrum avoidance among mothers having children aged 6–59 months in North Wollo Zone, Ethiopia. The findings of the present study are essential to develop evidence-based specific nutrition intervention for colostrum avoidance primarily in the study setting and also throughout the country with similar socioeconomic characteristics as a whole.

## Methods

### Study area and design

The North Wollo Zone is located at 521 km far from Addis Ababa and 251⋅75 km from the capital city of regional state called Bahirdar. North Wollo is one of eleven zones of the Amhara Region of Northern Ethiopia. South Wollo borders it on the south, on the west by South Gondar, on the north by Wag Hemra, on the Northeast by Tigray Region and on the east by Afar Region; part of its southern border is defined by the Mille River. Weldiya (also spelled Woldia). North Wollo acquired its name from the former province of Wollo. It is subdivided into 14 Woredas and 312 kebeles (276 rural and urban 36). The climatic condition of the Zone has varied between 10 and 27°C. Cereals are the staple foods. Most of this Zone is mountainous and characterised by steep slopes, which are unsuitable for agriculture and severely limits the cultivated area. An average farmland shared by a household is less than or equal to 0⋅7 hectare. Based on the national census conducted by the Central Statistical Agency of Ethiopia (CSA), this Zone has a total population of 1 788 901, of whom 895 189 are male and 893, 712 female; with an area of 12 172.50 km^2^. Of these, 241 502 are children aged 6–59 months. The governmental health institution of the Zone has 6 public hospitals, 68 health centres and 296 functional health posts.

### Study design and period

An institution-based cross-sectional study was conducted among mother–child pair aged 6–59 months attending under five child health services in the North Wollo Zone from 01 November to 24 December 2020.

### Source and study population

All mother–child pair aged 6–59 months who attends under five children in public health services at the North Wollo Zonal were taken as source population. All selected mother–child pair aged 6–59 months who attend under five children in public health services in the North Wollo Zonal until the end of data collection were the study population.

### Inclusion criteria

All mother–child pair aged 6–59 months coming for under five children public health services at the North Wollo Zonal during data collection was included.

### Exclusion criteria

Non-biological and mothers who were involuntary, severely ill and unable to communicate were excluded from the study during data collection.

### Sample size determination, sampling technique and sampling procedure

The minimum sample size was calculated using a single population proportion formula considering 95 % Confidence Interval (CI), 5 % margin of error, the proportion of colostrum avoidance 13⋅2 %^(20)^, design effect 2 and none response allowance 10 %. Finally, based on the assumptions, the required minimum sample size was taken as 388. A multi-stage sampling technique was employed. The North Wollo Zonal health institutions were selected purposively. The selected zone had a total of six public hospitals and 68 health centres which were stratified into hospitals and health centres. Among the existed zonal health institutions, one hospital and nine health centres were selected randomly with a lottery method. The first patient flow in the preceding 6 months was revised from the ANC registration book to compile the sampling frame. The sample size was then allocated based on population proportional to the size of under five children of each selected health facilities (one hospital and nine health centres) ANC registration book. Finally, the study units were selected from each health facilities ANC registration book by using a simple random sampling method. Data on mother–child pair were collected from mothers by reviewing ANC registration and family folder (for addition information) when they came to the selected facility for healthcare services at under five child healthcare units.

### Data collection tool and quality control

The questionnaire was consisting of socio-demographic characteristics, maternal and child healthcare practice. Data were collected using a 10 % of study subjects’ pre-tested interviewer-administered semi-structured questionnaire through a critical review of relevant literature. The questionnaire was prepared first in English and translated into Amharic (the local and national language), then back to English to check for consistency. The Amharic version of the questionnaire was used to collect the data. Eleven master of general public health students who can speak the local language were recruited as data collectors after 2 d training. Informed consent was obtained from all mothers. All women were interviewed personally with the help of a fixed questionnaire, regarding their feeding practices. When mother–child pair found having two or more eligible children, one of them was selected randomly via the lottery method.

### Study variable

The dependent variable was colostrum avoidance. The dependent variable was categorised into two: that mother–child pair whom were deprived of colostrum to the infant after delivery coded as ‘1’ and those who received colostrum coded as ‘0’ for regression analysis. The independent variables were maternal characteristics such as age, occupation, educational status, marital status and religion. Household characteristics like family size, household head for decision making and paternal educational status. Obstetric characteristics such as antenatal care (ANC), place of delivery, postnatal care (PNC) and mode of deliver. Child characteristics (sex, age), child feeding practices (colostrum feeding, pre-lacteal feeding, breast-feeding initiation and ever breast-feeding) and child feeding advice at ANC and PNC follow-up.

### Data processing and analysis

Data were checked for completeness and inconsistencies. It was also cleaned, coded and entered into Epi data version 4.6.02 software. Then, statistical product and service solutions (SPSS) version 25 was used to analyse the data. Descriptive statistics were estimated for continuous variables while frequency distribution was used to express the distribution of categorical variables and to show the prevalence of colostrum avoidance. Binary logistic regression analysis was performed. The crude odds ratio (COR) with a 95 % CI was estimated to assess the association between each independent variables and colostrum avoidance. Variables with a *P*-value < 0⋅25 in the binary logistic regression analysis were considered in the multivariable logistic analysis. The Hosmer-Lemeshow goodness-of-fit with enter procedure was used to test for model fitness. Adjusted odds ratio (AOR) with a 95 % CI was estimated to assess the strength of the association. Variables with a *P*-value < 0⋅05 in the multivariable logistic regression analysis were considered statistically significant and independent predictors of colostrum avoidance.

### Ethical approval

The study was approved by the Institutional Review Board (IRB) of Woldia University (Reference number: WDU/IRB/005/20; dated: 12 April 2020). The participants enrolled in the study were informed about the study objectives, expected outcomes, benefits and the risks associated with it. Consent was taken from the participants before the interview and measurement. Confidentiality of responses was maintained throughout the study.

### Operational definition

- *Colostrum avoidance:* This is a dependent variable which was categorised into two, those mother–child pair whom were deprived of colostrum to the infant after delivery coded as ‘1’ and those who received colostrum coded as ‘0’ for regression analysis^(11,18,21)^.
- *Pre-lacteal feeding:* defined as providing foods and/or drink other than human milk for the infant before breast-feeding was established^(12,22)^.
- *Breast-feeding initiation:* are the proportion of children born in the last 59 month who were made to breast-feed within 1 h of birth^(21,22)^.

## Result

### Socio-demographic characteristics of study participants

A total of 385 mothers having children aged under 5 years were included in the present study with 99⋅2 % response rate. Nearly two-third 236 (61⋅3 %) were orthodox by religion and almost all respondents 378 (98⋅2 %) belongs to Amhara by ethnicity. The majority of respondents were married 363 (94⋅3 %). More than half 219 (56⋅9 %) of the mothers were found in the age group of 25–34 years. The mean (±sd) age of mothers was 29⋅2 (±6⋅1) and ranged from 15 to 49 years. More than one-third 133 (34⋅5 %) of mothers were illiterate. Nearly one-third 130 (33⋅8 %) of the spouses/husbands of them also did not attend any school for education (Table 1).

**Table 1.** Socio-demographic characteristics of mother–child pair aged 6–59 months in North Wollo Zone, Amhara Region, Ethiopia, 2021 (*n* 385)

Variables	Category	Colostrum avoidance practice		Total *n* (%)
		No *n* (%)	Yes *n* (%)	
Maternal age	15–24	59 (15⋅3)	22 (5⋅7)	81 (21⋅0)
	25–34	187 (48⋅6)	32 (8⋅3)	219 (56⋅9)
	35 and above	66 (17⋅1)	19 (4⋅9)	85 (22⋅1)
	Mean (±sd)	29⋅2(±6⋅1)		
Ethnicity	Amhara	310 (80⋅5)	68 (17⋅7)	378 (98⋅2)
	Others^a^	2 (0⋅5)	10 (1⋅3)	7 (1⋅8)
Religion	Others^b^	118 (30⋅6)	31 (8⋅1)	149 (38⋅7)
	Orthodox	194 (50⋅4)	42 (10⋅9)	236 (61⋅3)
Marital status	Married	305 (79⋅2)	58 (15⋅1)	363 (94⋅3)
	Others^c^	7 (1⋅8)	15 (3⋅9)	22 (5⋅7)
Maternal education	Illiterate	102 (26⋅5)	31 (8⋅1)	133 (34⋅5)
	Read and write	18 (4⋅7)	13 (3⋅4)	31 (8⋅1)
	Primary	85 (22⋅1)	19 (4⋅9)	104 (27)
	Secondary and above	107 (27⋅8)	10 (2⋅6)	117 (30⋅4)
Maternal occupation	Housewife	199 (51⋅7)	54 (14)	253 (65⋅7)
	Others^d^	113 (29⋅4)	19 (4⋅9)	132 (34⋅3)
Paternal education	Illiterate	85 (22⋅1)	45 (11⋅7)	130 (33⋅8)
	Read and write	49 (12⋅7)	12 (3⋅1)	61 (15⋅8)
	Primary	46 (11⋅9)	6 (1⋅6)	52 (13⋅5)
	Secondary and above	132 (34⋅3)	10 (2⋅6)	142 (36⋅9)
Family size	≤4	168 (43⋅6)	39 (10⋅1)	207 (53⋅8)
	>4	144 (37⋅4)	34 (8⋅8)	178 (46⋅2)
	Mean (±sd)	4⋅6(±1⋅6)		
Household decision about child feeding practice	Paternal	280 (72⋅7)	46 (11⋅9)	326 (84⋅7)
	Maternal and paternal	32 (8⋅3)	27 (7)	59 (15⋅3)
Radio	Yes	190 (49⋅4)	41 (10⋅6)	231 (60⋅0)
	No	122 (31⋅7)	32 (8⋅3)	154 (40⋅0)
Television	Yes	137 (35⋅6)	39 (10⋅1)	176 (45⋅7)
	No	175 (45⋅5)	34 (8⋅8)	209 (54⋅3)

a Tigray, Oromia.

b Protestant, Muslim.

c Single, divorced and widowed.

d Governmental, NGOs, and daily labour.

### Maternal health and child care characteristics

Half of 196 (50⋅9) the mothers attended at least four and above ANC visit but one-tenth 39 (10⋅1 %) of them did not attend ANC follow-up. Of all study participants, one-fourth (20⋅8 %) of mothers were delivered their index child at their home. Regarding about maternal mode of delivery about 34 (8⋅8 %) were caesarean section (Table 2). About 361 (93⋅8 %) children aged 6–59 months were still on breast-feed. About 194 (50⋅4) child got the main source of food was from their own family production. Nearly one-fourth 98 (25⋅5) of children were exposed for pre-lacteal feeding practice (Table 3).

**Table 2.** Maternal health-related characteristics among mother–child pair aged 6–59 months in North Wollo Zone, Amhara Region, Ethiopia, 2021 (*n* 385)

Variables	Category	Colostrum avoidance		Total *n* (%)
		No *n* (%)	Yes *n* (%)	
Antenatal care follow-up	No	20 (5⋅2)	19 (4⋅9)	39 (10⋅1)
	1–3	125 (32⋅5)	25 (6⋅5)	150 (39)
	≥4	167 (43⋅4)	29 (7⋅5)	196 (50⋅9)
Place of delivery	Home	45 (11⋅7)	35 (9⋅1)	80 (20⋅8)
	Health institution	267 (69⋅4)	38 (9⋅9)	305 (79⋅2)
Mode of delivery	Caesarean section	29 (7⋅5)	5 (1⋅3)	34 (8⋅8)
	Vaginal	283 (73⋅5)	68 (17⋅7)	351 (91⋅2)
Postnatal care	No	129 (33⋅2)	33 (8⋅6)	161 (41⋅8)
	Yes	184 (47⋅8)	40 (10⋅4)	224 (58⋅2)
Birth order	First	110 (28⋅6)	25 (6⋅5)	135 (35⋅1)
	Second	93 (24⋅2)	14 (3⋅6)	107 (27⋅8)
	Third and above	109 (28⋅3)	34 (8⋅8)	143 (37⋅1)
Maternal nutrition status	MUAC below 23 cm	118 (30⋅6)	29 (7⋅8)	147 (38⋅2)
	MUAC 23 cm and above	194 (50⋅4)	44 (11⋅4)	238 (61⋅8)

**Table 3.** Child feeding-related characteristics among mother–child pair aged 6–59 months in North Wollo Zone, Amhara Region, Ethiopia, 2021 (*n* 385)

Variables	Category	Colostrum avoidance		Total *n* (%)
		No *n* (%)	Yes *n* (%)	
Initiation of breast-feeding	Early	208 (54)	16 (4⋅2)	224 (58⋅2)
	Late	104 (27)	57 (14⋅8)	161 (41⋅8)
Breast-feeding this child	No	0 (0)	24 (6⋅2)	24 (6⋅2)
	Yes	312 (81)	49 (12⋅7)	361 (93⋅8)
Pre-lacteal feeding	No	254 (66⋅0)	33 (8⋅6)	287 (74⋅5)
	Yes	58 (15⋅1)	40 (10⋅4)	98 (25⋅5)
Main source of foods	Own production	140 (36⋅4)	54 (14)	194 (50⋅4)
	Purchase	168 (43⋅6)	16 (4⋅2)	184 (47⋅8)
	Food donation/aid	4 (1⋅0)	3 (0⋅8)	7 (1⋅8)

### Prevalence of colostrum avoidance

Colostrum avoidance was 19 % (95 % CI 15⋅03, 22⋅89 %) among mother–child pair aged 6–59 months. From the total of 378 (98⋅2 %) Amhara ethnic group, study participants about 68 (17⋅7 %) were avoid the colostrum. Regarding from Orthodox religious fellow among 236 (61⋅3 %) mother–child pair about one-tenth 42 (10⋅9 %) of them were deprived of colostrum. From the total of 305 (79⋅2), mother–child pair aged 6–59 months attended institutional delivery about 38 (9⋅9 %) avoid colostrum. Among the total, 361 (93⋅8 %) of mother–child pair breast-feed children about 49 (12⋅7 %) were avoid the colostrum. From more than half of 207 (53⋅8), the respondents with four and below family size in the household about one-tenth 39 (10⋅1 %) were avoid the colostrum. Among one-fifth 80 (20⋅8 %) of respondents who delivered at home nearly one-tenth 35 (9⋅1 %) of them were avoid colostrum (Tables 1–3).

### Predictor of colostrum avoidance practice

Variables such as mode of delivery, radio, family size and maternal nutritional status were not eligible for colostrum avoidance during the statistical testing process in binary logistic regression analysis. The variables like paternal education, maternal education, maternal age, ANC visit and TV were associated with the dependent variable in the bi-variable regression analysis, but they failed to maintain their association consistently with the dependent variable in the multivariable logistic regression analysis. Finally, the multivariable analysis identified pre-lacteal feeding AOR 3⋅464; 95 % CI (1⋅721, 6⋅973), household decision about child feeding AOR 3⋅585; 95 % CI (1⋅563, 7⋅226), Index child sex AOR 2⋅103; 95 % CI (1⋅015, 4⋅358) and health facility delivery AOR 3⋅033; 95 % CI (1⋅293, 7⋅117) as associated factors for colostrum avoidance (Table 4).

**Table 4.** Predictors of colostrum avoidance among mother–child pair aged 6–59 months in North Wollo Zone, Amhara Region, Ethiopia, 2021 (*n* 385)

Variables/category	Colostrum avoidance		COR (95 % CI)	AOR (95 % CI)
	No	Yes		
Paternal education				
Illiterate	85	45	6⋅988 (3⋅343, 14⋅609)	2⋅919 (0⋅927, 9⋅190)
Read and write	49	12	3⋅233 (1⋅313, 7⋅959)	1⋅046 (0⋅279, 3⋅919)
Primary	46	6	1⋅722 (0⋅593, 5⋅001)	0⋅827 (0⋅192, 3⋅559)
Secondary and above	132	10	1	1
Maternal education				
Illiterate	102	31	3⋅252 (1⋅517, 6⋅972)	0⋅877 (0⋅267, 2⋅888)
Read and write	18	13	7⋅728 (2⋅945, 20⋅260)	2⋅983 (0⋅720, 12⋅354)
Primary	85	19	2⋅392 (1⋅057, 5⋅414)	1⋅414 (0⋅457, 4⋅379)
Secondary and above	107	10	1	1
Household decision about child feeding practice				
Maternal and paternal	32	27	1	1
Paternal	280	46	5⋅136 (2⋅820, 9⋅355)	**3⋅585** (1⋅563, 7⋅226)***
Index child sex				
Female	147	23	1⋅937 (1⋅127, 3⋅329)	**2⋅103** (1⋅015, 4⋅358)***
Male	165	50	1	1
TV				
No	137	39	1	1
Yes	175	34	1⋅465 (0⋅879, 2⋅443)	1⋅010 (0⋅444, 2⋅298)
Maternal age				
15–24	59	22	1⋅925 (0⋅639, 2⋅627)	1⋅873 (0⋅699, 5⋅018)
25–34	187	32	0⋅597 (0⋅316, 1⋅120)	0⋅769 (0⋅326, 1⋅813)
35 and above	66	19	1	1
Antenatal care visit				
No	20	19	5⋅471 (2⋅606, 11⋅483)	0⋅752 (0⋅239, 2⋅359)
1–3	125	25	1⋅152 (0⋅642, 2⋅063)	0⋅715 (0⋅330, 1⋅548)
≥4	167	29	1	1
Place of delivery				
Home	45	35	5⋅465 (3⋅130, 9⋅542)	**3⋅033** (1⋅293, 7⋅117)***
Health institution	267	38	1	1
Breast-feeding initiation				
Early	208	16	1	1
Late	104	57	7⋅125 (3⋅901, 13⋅014)	6⋅369 (3⋅067, 13⋅224)*
Pre-lacteal feeding	254	33	1	1
	58	40	5⋅308 (3⋅087, 9⋅128)	3⋅464 (1⋅721, 6⋅973)**

* *P* < 0⋅0001, **P < 0⋅001, ***P < 0⋅005, ****P < 0⋅05.

## Discussion

Colostrum is still discarded from different parts of the globe particularly in developing country in Ethiopia^(14,18)^. The present study was conducted to assess the level of colostrum avoidance and its predictors among mother–child pair aged 6–59 months in North Wollo Zone, Amhara Region, Ethiopia.

It was slightly similar with systematic review and meta-analysis database studies revealed in Motta 20⋅3 %^(23)^. The finding was almost similar to another study revealed from Bure district 14⋅5 %^(11)^, Gozamen district 22⋅1 %^(24)^. It was higher than the previous studies revealed from different parts of Ethiopia such as 12 %^(19)^, Aksum town 6⋅3 %^(14)^, Bahirdar city 8⋅8 %^(25)^, Derbretabor 10⋅5 %^(26)^ and Kombolcha town 11⋅5 %^(18)^. Studies conducted in African countries such as Cameroon 11⋅2 %^(16)^ and Bhutan 13⋅2 %^(20)^ had lower colostrum avoidance as compared to this finding.

On the other hand, the present study was lower than other studies revealed from Debre tabor 25⋅6 %^(13)^, Afar 76⋅9 %^(27)^, Jima Arjo district 27⋅5 %^(28)^. It was also lower than outside Ethiopian country such as Rural Pakistan 27⋅9 %^(29)^, Block RS Pura 76 %^(30)^ and Gujarat India 29⋅5 %^(10)^. The possible explanation for this observed discrepancy might be the fact that the study population, socio-demographic characteristics, socio-cultural characteristics, study design and different study setting.

According to the present study, place of delivery, breast-feeding initiation, decision making, pre-lacteal feeding and index child sex preferences are predictor variables for colostrum avoidance.

In the present study, regarding factors associated with colostrum avoidance, mothers having late initiation of breast-feeding were 6⋅369 times more likely to discard colostrum as compared with their counterparts. This is consistent with studies revealed in Kombolcha district^(18)^, pastoralist Afar community^(27)^, Bahirdar City^(25)^. This might be also indicating the presence of poor nutrition-specific intervention mothers who did not get counselling about timely imitation of breast-feeding^(18,24,31)^. It might be also due to the fact that absence of ANC follow-up, poor knowledge^(31)^, elders and or relative advice^(10)^ and poor attitude^(11,18,24)^.

Mothers attended home deliveries were depriving their children from colostrum feeding more than three times than those who delivered at health institutions. This finding was consistent with studies revealed from Gozamen district^(24)^, Wolaita Sodo town^(32)^, India^(10)^. This finding was also consistent with studies revealed from the previous study of this area^(19)^. This might be due to the fact that lack of receiving ANC counselling about optimal breast-feeding practice^(18,24,26,32,33)^.

The present study showed that mothers who gave pre-lacteal feeding to their children were 3⋅46 times more likely to discard colostrum as compared with those who had the colostrum for their children. This was supported by studies revealed from Northern parts of Ethiopia^(19,24)^. The possible explanation might be due to the fact that mothers might not participating in pregnant mothers forum^(18)^, inadequate follow-up visit for ANC and PNC visit, poor maternal level of information about colostrum^(14,34)^.

Paternal household decision about child feeding had 3⋅58 times more likely for discarding colostrum as compared with those who were deciding together both husband and mother to their children feeding practice. This is supported by findings from nationally representative data from Ghana women's participation in household decision-making and higher dietary diversity^(35)^.

Being female index child had 2⋅1 times more likely for discarding colostrum as compared with those mothers who had children with sex being male. This is supported by different studies revealed from Ethiopia and outside Ethiopia such as Cameroon, India^(16)^. This might be also due to the deep-rooted cultural influences, ethnic background and a range of socio-demographic factors. These were more likely to influence the roles of mothers and fathers in child rearing, feeding and ensuring diversity in parents participating in interventions that will be critical to building a robust evidence base for the role of early feeding practices of both parents^(36)^.

### Limitations of the study

Recall bias about colostrum avoidance was one of the limitations. The lack of qualitative data supplementation especially to explore deep-rooted cultural believes why the community practiced the colostrum avoidance and how to reduce it through targeted participants of mothers, health workers and key influential leaders in the community. It also shares the limitation of cross-sectional study design.

## Conclusion

The present study revealed that 19 % of mother–child pair avoids the colostrum. Place of delivery, late breast-feeding initiation, paternal decision-making, pre-lacteal feeding and sex preferences were the most important predictors.

Therefore, it should be strengthening adolescent maternal–infant young child nutrition policy through counselling about the significant role of mothers for optimal child development to curve and correct sex preferences.

The shared household decision about colostrum feeding through the promotion of the free home delivery policy of Ethiopia is significantly important to reduce colostrum avoidance.

Finally, the scientific community should study with a prospective cohort study design including with supplementation of qualitative data to identify other potential independent predictors.

## References

[1] Silva P (2005) *Environmental Factors and Children's Malnutrition in Ethiopia*. Policy Research Working Paper; No. 3489. Washington, DC: World Bank. © World Bank. https://openknowledgeworldbankorg/handle/10986/8898 License: CC BY 30 IGO.

[2] World Health Organization UNICEF (2003) Global Strategy for Infant and Young Child Feeding. https://www.who.int/nutrition/publications/gs_infant_feeding_text_eng.pdf.

[3] SUN Movement's Newsletter (2018) Breastfeeding: A Mothers Gift for Every Child. https://scalingupnutrition.org/news/breastfeeding-a-mothers-gift-for-every-child/.

[4] UNICEF WHO Implementation Guidance (2020) Protecting, Promoting and Supporting Breastfeeding: The Baby-Friendly Hospital Initiative for Small, Sick and Preterm Newborns. https://www.babyfriendly.org.nz/fileadmin/Documents/Protecting_promoting_and_supporting_breastfeeding_NICU_WHO2020.pdf 3.0 IGO Licence (CC BY-NC-SA 3.0 IGO).

[5] Gebrehiwot H, Thampi A, Kassaw Y, (2018) Knowledge, attitude and practice towards colostrum feeding among antenatal care attendant pregnant women in Mekelle health facilities, Mekelle, Tigray, Ethiopia, 2018. Int J Dev Res 8, 24836–24841.

[6] WHO, UNICEF (2009) World Bank, State of the World's Vaccines and Immunization, pp. 130–145. Geneva: World Health Organization.

[7] Victora CG, Bahl R, Barros AJ, (2016) Breastfeeding in the 21st century: epidemiology, mechanisms, and lifelong effect. The Lancet 387, 475–490. 10.1016/S0140-6736(15)01024-7 26869575

[8] Gupta A, Holla R, Dadhich J, (2013) The status of policy and programmes on infant and young child feeding in 40 countries. Health Policy Plann 28, 279–298. 10.1093/heapol/czs061 22763127

[9] Dhale P & Mahakalkar M (2017) A study to assess the knowledge and attitude regarding importance of colostrum among postnatal mothers in selected hospitals. Int J Sci Res 6, 2239–2241.

[10] Kakati R, Rahman SJ, Borah M, (2016) Colostrum feeding practices and its determinants among urban and rural mothers in Kamrup, Assam, India. Int J Res Med Sci 4, 4567–4572.

[11] Mose A, Dheresa M, Mengistie B, (2021) Colostrum avoidance practice and associated factors among mothers of children aged less than six months in Bure District, Amhara Region, North West, Ethiopia: a community-based cross-sectional study. PLoS ONE 16, e0245233. 33513154 10.1371/journal.pone.0245233 PMC7846012

[12] Bililign N, Kumsa H, Mulugeta M, (2016) Factors associated with prelacteal feeding in north eastern Ethiopia: a community based cross-sectional study. Int Breastfeed J 11, 1–7. 27190547 10.1186/s13006-016-0073-x PMC4869312

[13] Abie BM & Goshu YA (2019) Early initiation of breastfeeding and colostrum feeding among mothers of children aged less than 24 months in Debre Tabor, northwest Ethiopia: a cross-sectional study. BMC Res Notes 12, 1–6. 30696481 10.1186/s13104-019-4094-6 PMC6352422

[14] Weldesamuel GT, Atalay HT, Zemichael TM, (2018) Colostrum avoidance and associated factors among mothers having children less than 2 years of age in Aksum town, Tigray, Ethiopia: a cross-sectional study 2017. BMC Res Notes 11, 1–7. 30126446 10.1186/s13104-018-3712-z PMC6102877

[15] Joshi SK, Barakoti B, Lamsal S (2012) Colostrum feeding: knowledge, attitude and practice in pregnant women in a teaching hospital in Nepal. Int J Med Mol Med. https://www.webmedcentral.com/wmcpdf/Article_WMC003601.pdf.

[16] Tambe BA, Mimboe C, Nchung JA, (2018) The determinants of exclusive breastfeeding in Cameroon, Sub-Saharan Africa. Trends General Pract 1, 2–6.

[17] Gualu T, Adugna H & Dilie A (2017) Assessment of knowledge, attitude and practice of post natal mothers towards colostrum breast milk in Debre Markos town governmental health institutions east Gojjam zone, Amhara regional state, Ethiopia. Nurs Care Open Access J 2, 55–60.

[18] Gebreyesus H, Girma E & Cherie N (2017) Colostrum avoidance and associated factors among mothers of children aged less than 12 months in Kombolcha town, South Wollo zone, Ethiopia. Medico Res Chronicles 4, 545–559.

[19] Yimer NB & Liben ML (2018) Effects of home delivery on colostrum avoidance practices in North Wollo zone, an urban setting, Ethiopia: a cross sectional study. J Health, Popul Nutr 37, 1–7. 29482631 10.1186/s41043-018-0134-4 PMC6389058

[20] Tshering D, Gurung MS, Wangmo N, (2019) Knowledge attitude and practice of exclusive breastfeeding among breastfeeding mothers in Trongsa district, Bhutan. Bhutan Health J 5, 21–25.

[21] World Health Organization (2009) Infant and Young Child Feeding: Model Chapter for Textbooks for Medical Students and Allied Health Professionals. Geneva: WHO; available at https://apps.who.int/iris/bitstream/handle/10665/44117/9789241597494_eng.pdf?sequence=1&isAllowed=y. 23905206

[22] Argaw MD, Asfaw MM, Ayalew MB, (2019) Factors associated with prelacteal feeding practices in Debre Berhan district, North Shoa, Central Ethiopia: a cross-sectional, community-based study. BMC Nutr 5, 1–9. 32153927 10.1186/s40795-019-0277-8 PMC7050708

[23] Tewabe T, Mandesh A, Gualu T, (2016) Exclusive breastfeeding practice and associated factors among mothers in Motta town, East Gojjam zone, Amhara Regional State, Ethiopia, 2015: a cross-sectional study. Int Breastfeed J 12, 1–7. 28261318 10.1186/s13006-017-0103-3 PMC5327553

[24] Azene ZN, Mulunesh A & Alamneh TS (2021) Delayed breast feeding initiation increases the odds of colostrum avoidance among mothers in Northwest Ethiopia: a community-based cross-sectional study. Arch Public Health 79, 1–11. 33827691 10.1186/s13690-021-00571-x PMC8028159

[25] Ayalew T, Asmare E (2021) Colostrum avoidance practice among primipara mothers in urban Northwest Ethiopia. A cross-sectional study. BMC Pregnancy Childbirth 21, 1–9. 33573612 10.1186/s12884-021-03623-w PMC7879506

[26] Addisu D, Melkie A, Bezie M, (2020) Determinants of colostrum avoidance among postpartum mothers in North West Ethiopia. J Midwifery Reprod Health 8, 2504–2511.

[27] Gebretsadik GG, Tkuwab H, (2020) Early initiation of breastfeeding, colostrum avoidance, and their associated factors among mothers with under one year old children in rural pastoralist communities of Afar, Northeast Ethiopia: a cross sectional study. BMC Pregnancy Childbirth 20, 1–9. 10.1186/s12884-020-03151-z PMC7405449 32758166

[28] Tamiru D, Belachew T, Loha E, (2012) Sub-optimal breastfeeding of infants during the first six months and associated factors in rural communities of Jimma Arjo Woreda, Southwest Ethiopia. BMC Public Health 12, 1–9. 22607266 10.1186/1471-2458-12-363 PMC3439284

[29] Sohail J & Khaliq A (2017) Knowledge, attitude, and practice of mothers regarding colostrum feeding to newborns in rural Pakistan: a cross-sectional study. Khyber Medical Univ J 9, 192–196. 5p.

[30] Raina SK, Mengi V & Singh G (2012) Differentials in colostrum feeding among lactating women of block RS Pura of J and K: a lesson for nursing practice. Iran J Nurs Midwifery Res 17, 386. 23853653 PMC3703081

[31] Hadona EA, Weldehawariat FG, Sorrie MB (2020) Colostrum avoidance practices and its associated factors among mothers of children aged less than 12 months in Jinka Town, South Ethiopia, 2019, 1–15. Available from DOI: 10.21203/rs.3.rs-48987/v1.

[32] Gargamo DB (2020) Colostrum feeding practices and associated factors among mothers having children less than 12 months of age in Wolaita Sodo City, Wolaita, Ethiopia 2019. Biomed Sci 6, 17.

[33] Yeshambel Wassie A, Atnafu Gebeyehu N & Abebe Gelaw K (2020) Knowledge, attitude, and associated factors towards colostrum feeding among antenatal care attendant mothers in Gununo Health Centre, Wolaita Zone, Ethiopia 2019: across-sectional study. Int J Pediatr 2020, 3453502, 1–10. 10.1155/2020/3453502 PMC6996677 32099549

[34] Setegn T, Belachew T, Gerbaba M, (2012) Factors associated with exclusive breastfeeding practices among mothers in Goba district, south east Ethiopia: a cross-sectional study. Int Breastfeed J 7, 1–8. 23186223 10.1186/1746-4358-7-17 PMC3560275

[35] Amugsi DA, Lartey A, Kimani-Murage E, (2016) Women's participation in household decision-making and higher dietary diversity: findings from nationally representative data from Ghana. J Health Popul Nutr 35, 1–8. 27245827 10.1186/s41043-016-0053-1 PMC5026004

[36] Daniels LA (2019) Feeding practices and parenting: a pathway to child health and family happiness. Ann Nutr Metab 74, 29–42. 31234189 10.1159/000499145

